# Create a translational medicine knowledge repository - Research downsizing, mergers and increased outsourcing have reduced the depth of in-house translational medicine expertise and institutional memory at many pharmaceutical and biotech companies: how will they avoid relearning old lessons?

**DOI:** 10.1186/1479-5876-9-56

**Published:** 2011-05-10

**Authors:** Bruce H Littman, Francesco M Marincola

**Affiliations:** 1 Translational Medicine Associates, L.L.C., Stonington, CT 06378, USA; 2 Infectious Disease and Immunogenetics Section (IDIS)-Department of Transfusion Medicine, Clinical Center, National Institutes of Health, Bethesda, MD 20892, USA

## Abstract

Pharmaceutical industry consolidation and overall research downsizing threatens the ability of companies to benefit from their previous investments in translational research as key leaders with the most knowledge of the successful use of biomarkers and translational pharmacology models are laid off or accept their severance packages. Two recently published books may help to preserve this type of knowledge but much of this type of information is not in the public domain. Here we propose the creation of a translational medicine knowledge repository where companies can submit their translational research data and access similar data from other companies in a precompetitive environment. This searchable repository would become an invaluable resource for translational scientists and drug developers that could speed and reduce the cost of new drug development.

## 

There is a well known problem in big pharma, low productivity despite high costs. It has become clear that the pharmaceutical industry's business model is broken [1]. Research-based large pharmaceutical companies have failed to fill their pipelines with enough successful new drugs to maintain growth and replace revenues from older products going off patent. Instead their pipelines have been filled with drug projects that largely have failed to make it to market [2]. Many reasons for this have been postulated but here we focus on the responses of companies to this issue and the repercussions of those actions for translational research in the industry.

Companies have consolidated through mergers and acquisitions resulting in lay-offs for thousands of researchers and the closing of hundreds of laboratories to avoid perceived redundancies, focus on what is considered to be more productive or lucrative therapeutic areas and save costs [3]. Just consider the closing of Pfizer's Ann Arbor and St. Louis research sites, the recently announced moving of drug discovery operations from their largest site in Groton, CT and the announced closure of their Sandwich Laboratories in the U.K. These four sites were involved in the demise of many drug projects due to very high rates of attrition but they were also involved in the discovery and development of many great drugs over the last 25 years. Successful branded drugs discovered and/or developed at these sites (in alphabetical order) include Aricept, Cardura, Celebrex, Chantix, Diflucan, Feldene, Geodon, Glucotrol, Lipitor, Lyrica, Neurontin, Norvasc, Procardia, Selzentry, Tarceva (achieved proof of concept at Groton Labs before its required divestiture), Viagra, Zithromax, Zoloft and Zyrtec to name just some. Consider the lessons learned from both the failures and the successes. Pfizer was one of the first companies to embrace the field of experimental medicine and then translational medicine as specialized functions during the drug discovery and early drug development process [4]. They invested millions of dollars in biomarker development and the qualification of human pharmacology models to enable data-driven decisions for drugs with novel targets but these were often not put into the public domain. Now consider the loss of institutional memory as leaders in these areas left the company either as a result of early retirement or attractive severance packages as downsizing and consolidation ruled. This also occurred at many other companies including Merck, Lilly, Roche and GSK [5]. Many of the hard won lessions of translational research and early drug development could well be lost and the more recent successful strategies for coping with high attrition rates may have to be relearned.

One of the reasons Littman and Krishna decided to create a textbook entitled ***Translational Medicine and Drug Discovery ***was to provide a guidebook for current and future translational medicine scientists and to clearly describe successful strategies for early drug development that utilize biomarkers and state of the art technologies. This book partially addresses the issue described above. It includes sections and chapters describing successful translational strategies for early drug development, principles of biomarker qualification and utilization in drug discovery and development, in-depth examples of translational research in six different therapeutic areas, translational imaging technologies, modeling and simulation and examples of pre-competitive collaborations between companies in many areas including biomarkers and drug safety [6]. Another similar book, ***Biomarkers in Drug Development***, also attempts to serve as a "handbook of practice, application and strategy" for translational researchers [7]. However, textbooks like these cannot do the job alone and they are static. They are not regularly updated, newer methods and biomarkers are not added and comparisons of results between different drugs with similar mechanisms cannot be made.

We believe that companies and other institutions involved in translational drug research need to foster the discipline of translational medicine, maintain a knowledge base of their work and, when it is not proprietary, share that knowledge through publication and pre-competitive collaborations. It is often easier to find the published results of translational research performed by others than it is to find the unpublished results of translational research done within your own institution, especially if the drug projects the research supported were discontinued. For this reason we advocate the creation of a searchable knowledge repository for human translational research. This database could be maintained by a consortium of drug companies that voluntarily submit data and it would include human biomarker data, translatable human pharmacology models and other types of clinical methods that have been used in humans to measure drug responses. A structured database like this could be initially populated by data from failed drug projects with no significant loss of competitive advantage to contributing companies and institutions. Also, time is not on our side. The opportunities to capture this type of information decreases almost monthly as companies continue to reorganize, downsize and consolidate. It can only happen if those involved in these projects are still employed and have access to their companies' data.

Imagine the benefits if this human translational medicine database existed today and could be searched by drug target, pathway, biomarker, disease, therapeutic area, challenge agent and translational pharmacology model name or description. It would become the first place to look when developing a translational research plan for a new drug project. It could potentially prevent reinventing proven clinical methods and replicating past biomarker and human model qualification efforts. Often there are multiple choices for achieving proof of mechanism for new drugs and this database would simplify the choice when an acceptable existing method or biomarker can be easily found. Use of the database could also translate into reduced costs and reduced time for drug development and enable the comparison of results from newer drugs with those from past drug projects. These comparisons will also aid decision-making based on the data from existing projects compared to older failed or successful projects. We urge companies to strongly consider adopting this recommendation and finding a home for the database perhaps under the auspices of an existing consortium such as The Biomarker Consortium managed by the Foundation for the National Institutes of Health (FNIH) [8].
